# Risky sexual behaviors of schizophrenic patients: a single center study in Ethiopia, 2018

**DOI:** 10.1186/s13104-019-4673-6

**Published:** 2019-09-27

**Authors:** Biruk Negash, Bethlehem Asmamewu, Wondale Getinet Alemu

**Affiliations:** 1 Ministry of Health, Geferessa Rehabilitation Center, Addis Ababa Health Bureau, Addis Ababa, Ethiopia; 2 Ministry of Health, Amanuel Mental Specialized Hospital, Addis Ababa Health Bureau, Addis Ababa, Ethiopia; 3 Department of Psychiatry College of Medicine and Health Science, University of Gondar, Gondar, Ethiopia

**Keywords:** Risky sexual behavior, Schizophrenia, Psychosis, Ethiopia

## Abstract

**Objective:**

Identify factors related to risky sexual behavior can facilitate health care providers to approach programs that improve quality of services provided to the patient service. The aim of study to assess the prevalence of risky sexual behaviors and associated factors among schizophrenia patient at Amanuel Mental specialized hospital, Addis Ababa, Ethiopia, 2019.

**Result:**

A total of four hundred twenty-nine participants were interviewed with a response rate of 97.05%. The prevalence of risky sexual behavior was 39.4% (95% CI 34.3, 43.6). In the multivariate logistic regression, being male sex (AOR = 3.78 (1.94, 7.38)), patients in age group between 18 and 24 (AOR = 4.85 (1.73, 13.6)), current use of alcohol (AOR = 1.86 (1.049, 3.32)), place of residence (AOR = 6.22 (2.98, 12.98)), positive symptom (AOR = 3.01 (1.55, 5.84)) were associated with risky sexual behavior.

## Introduction

Risky sexual behavior is the description of the activity that increases the probability of a person engaging in sexual activity with another person infected with a sexually transmitted infections, will be infected or become pregnant, or make a partner pregnant. It involves negative consequence including unintended pregnancy and HIV/AIDS or other transmitted diseases, gained due to several sexual partners, inconsistent use of condom and having sexual intercourses under influence of alcohol [1].

People with schizophrenia are susceptible to risky sexual behavior [2]. Being sexually active is less common among them; those who are active are more likely to engage in risky sexual behavior [3]. Persons may experience “negative” symptoms which include loss of a sense of pleasure, social withdrawal, poverty of thoughts and speech, and flattening of affect and, medication side effects disrupt patient’s ability to perform sexually, however, these problems do not eliminate the desire for sexual contact or render the patient inactive [4, 5].

Several studies conducted among adults showed that there was an increasing risky sexual behavior people with schizophrenia at global disease burden, schizophrenia spectrum was two times more likely to engage in risky sexual intercourse and 2.3 times to develop transmitted diseases [6].

Among studies done on New York state 93% of them use condoms, 62% had multiple sexual partners, 50% took part in sex exchange, 45% of them used drugs or alcohol during sex [7], and other studies in New York, having multiple sexual partners were three times as likely among patients with greater positive symptom [7]. In the UK condom use was very inconsistent (around 8%), drug or alcohol use during sexual intercourse was common, and sexual exchange (for money, drugs or other goods), around 12% of those who were active had sex with a partner who was a known drug user [2]. In Nigeria 38.2% reported two or more sexual partners, 5.9% reported sex trading and 80.8% inconsistent use of condom [8], community-based cross-sectional study conducted in New York 62% had multiple sexual partners, 50% took part in sex exchange, 45% of the active patients used drugs or alcohol during sex, which may affect risk-taking behavior [5].

Cross-sectional study conducted on Italy stated that 83% of schizophrenia engage in high-risk sexual activity at which (58% had multiple sexual partners, 25% of them had almost never used a condom) [9]. A case–control study conducted on Turkey of risky sexual behaviors of among diagnosis of schizophrenia was 26%, such as sex with multiple sexual partners 6%, sexual intercourse with different partners 10%, sexual activity under influence of alcohol or other drugs 10% in participants [10] in India, 59% of had a history of risky sexual behavior [11], Nigeria (48%), of the prevalence of risky sexual behavior [8].

The estimated incidence of HIV/AIDS among people ranged from 4 to 23% [12], and 21% people with schizophrenia were following ART treatment [13].

So, determining the prevalence of risky sexual behavior and associated factors among schizophrenia patient is important for early intervention and the reduction of the burden of risky sexual behavior and to improve the quality of life of schizophrenia patients.

## Main text

### Study setting and population

An institution based cross-sectional study was conducted from May to June 2018 at Amanuel Mental Specialized Hospital, Addis Ababa, Ethiopia. Amanuel Mental Specialized hospital is a governmental hospital in Addis Ababa, which is found in Addis ketema sub city, the capital city of Ethiopia.

The study included participants aged 18 years and above during data collection in the area. There were a total of 3536 people with schizophrenia spectrum disorder receiving follow-up care at the outpatient department of Amanuel Mental Health Specialized Hospital. Individuals seriously ill and unable to communicate were excluded. We determined the sample size by using the single population proportion formula with the assumption of 50% prevalence of risky sexual behavior, 95% CI (alpha = 0.05), 1.96 standard normal distribution and a 10% non-response rate. Accordingly, a representative sample was calculated to be four hundred twenty-three. After considering non response rate the total sample size was four hundred forty-two. We selected the study participants using systematic random sampling technique.

### Data collection and analysis

Data were collected by face to face interviews, using a semi structured questionnaire by means of the Amharic version of the tool for a month. The questionnaire was designed in English and translated to Amharic and back to English to maintain consistency. Data collectors were trained on how to interview participants and explain unclear question and the purpose of the study. Furthermore, they were made aware about ethical principles, such as confidentiality/anonymity/data management and securing respondents informed consent for participation. Risky sexual behavior was measured using the adopted from behavioral surveillance survey and published articles which changed to apply for the local context. It was validated in Ethiopian demographic and health survey for assessment of risky sexual behavior among HIV/AIDS patients. Tools which consist of risky sexual behaviors (such as early sexual initiation, multiple sexual partners, unprotected sex, the use of substances and involved in sex [14, 15]). Positive and negative syndrome scale (PANSS) (a 30-item semi-structured interview) to assess positive, negative and global psychopathology symptoms. Before starting actual survey, the questionnaire was pre-tested on 5% of sample size at a St. Paul hospital. According to pretest Cronbach’s Alpha value was 0.67. Two days training was given for data collectors and supervisor on the content of the questionnaires. All collected data were checked for completeness, consistency and recheck before processing and analysis of the data. Then, entered into EPINFO 3.5.1 statistical software and exported to SPSS windows version-20 for analysis. We computed descriptive, bivariate and multivariate logistic regression analyses to see the frequency distribution and to test the association between independent and dependent variables, respectively. Factors associated with risky sexual behavior were selected during the bivariate analysis with a p-value < 0.2 for the analysis, variables with p-value less than 0.05 at 95% confidence interval with adjusted odds ratio were considered as statistically significant.

### Results

#### Socio demographic characteristics of participants

A total of 442 participants took part with a response rate of 97% (429). The mean age of respondents was 32.8 years (± SD = 8.9) Out of 429 respondents, 253 (59%) were males, 190 (44.3%) were orthodox religion followers, 234 (54.5%) were singles, 221 (51.5%) had secondary education, 213 (49.7%) were unemployed, 275 (64.1%) lived in towns. From the total 298 (69.5%) were developed schizophrenia before the age of 25 years, 170 (39.6%) of them had the illness for 5–10 years, about 173 (40.3%) were on treatment for 5–10 years, 280 (65.1%) had less than two episodes, 277 (64.6%) had history of hospitalization (Table 1).

**Table 1:** Socio-demographic and clinical characteristics of clients with schizophrenia on follow up at AMSH, Addis Ababa, Ethiopia, 2018 (N = 429)

Variables	Categories	Frequency	Percentage
Sex	Male	274	63.8
	Female	155	36.2
Age	18–24	77	17.9
	25–34	187	43.6
	35–44	123	28.7
	> 44	42	9.8
Religion	Orthodox	190	44.3
	Muslim	113	26.3
	Protestant	65	15.2
	Others	61	14.2
Marital status	Single	234	54.5
	Married	97	22.6
	Divorced	76	17.7
	Widowed	22	5.1
Educational status	Can’t read and write	29	6.8
	Primary	74	17.2
	Secondary	221	51.5
	College and above	105	24.5
Job	Had job	199	46.3
	Had no job	230	53.7
Place of residence	Urban	275	64.1
	Rural	154	35.9
Age at onset of the illness (years)	≤ 25	298	69.5
	> 25	131	30.5
Duration of the illness (years)	≤ 5	130	30.3
	5–10	170	39.6
	> 10	129	30.1
Duration of treatment (years)	≤ 5	153	35.7
	5–10	173	40.3
	> 10	103	24
Number of episodes	≤ Two	280	65.2
	> Two	149	34.8
Presence of hospitalization	Yes	277	64.6
	No	152	35.4
Number of hospitalization (N = 277)	Less than equal to 4	177	63.8
	Less than 4	100	36.2
Lifetime history of homelessness	Yes	96	22.4
	No	333	77.6
History of childhood sexual abuse	Yes	39	9.1
	No	390	90.9
History of adult sexual abuse	Yes	64	14.9
	No	365	85.1
PANNS	Less than equal to 56	285	66.4
	Less than 56	144	33.6
Positive symptom	Less than equal to 12	232	54.1
	Less than 12	197	45.9

#### Prevalence of risky sexual behavior among schizophrenic patients

The overall prevalence of risky sexual behavior was 39.4%, 95% CI (34.3, 43.6). Among all 8.4% had sexual intercourse with two or more sexual partners, 10.5% had unprotected sexual intercourse, 11.2% had sexual intercourse after alcohol consumption and; 12.1% had exchanged money for sex.

#### Factors associated with risky sexual behavior

To examine the association of independent variables with risky sexual behavior, bivariate and multivariate binary logistic regression analyses were done.

In the bivariate analyses, factors including, being male sex, being in age category of 18–24, age onset of illness, homelessness, urban residence, current use of tobacco, positive symptom and current use of alcohol were significantly associated with risky sexual behavior at a p-value less than 0.2. These factors were entered into the multivariable logistic regression model to control confounding effects. The result of the multivariate analysis showed that being male sex, being in age category of 18–24, urban residence, Current alcohol use, Positive symptom were significantly associated with risky sexual behavior at a p-value less than 0.05. Male sex was 3.78 times more likely to develop risky sexual behavior compared with female sex (AOR = 3.78, 95% CI 1.94, 7.38). The odds of developing risky sexual behavior were 4.85 times higher among age group 18–24 compared with the ones greater than/equal to 45 years (AOR = 4.85, 95% CI 1.73, 13.6). The odds of developing risky sexual behavior were 6.22 times higher among participants from urban residence compared with those who are from rural (AOR = 6.22, 95% CI 2.98, 12.98). The likelihood of developing risky sexual behavior was 1.86 times higher among respondents who are current alcohol users compared with those who are not alcohol users (AOR = 1.86, 95% CI 1.049, 3.32). The odds of developing risky sexual behavior were 3 times higher among respondents who have positive symptoms than those who hadn’t positive symptom (AOR = 3, 95% CI 1.55, 5.84) (Table 2).

**Table 2:** Factors associated with risky sexual behavior among clients with schizophrenia at outpatient clinic AMSH, Addis Ababa, Ethiopia, 2018 (N = 429)

Explanatory variables	Level	Risky behavior		COR (95% CI)	AOR (95% CI)
		Yes	No		
		N	N		
Sex	Male	147	127	1.849 (1.221, 2.80)**	3.78 (1.94, 7.38)***
	Female	22	133	1	1
Age	18–24	57	20	6.53 (2.93, 14.54)***	4.85 (1.73, 13.6) **
	25–34	52	135	1.493 (0.79, 2.86)	0.93 (0.404, 2.14)
	35–44	40	83	1.483 (0.75, 2.91)	0.95 (0.416, 2.21)
	≥ 45	13	29	1	1
Age onset of illness (years)	≤ 25	174	124	1.76 (1.19, 2.77)*	1.22 (0.61, 2.42)
	> 25	39	92	1	1
Homelessness	Yes	77	19	1.235 (0.146, 0.377)**	2.38 (0.986, 2.42)
	No	99	234	1	1
Residence	Urban	140	135	7.21 (4.33, 12.015)***	6.22 (2.98, 12.98)***
	Rural	36	118	1	1
Current alcohol use	Yes	128	94	4.34 (2.85, 6.62)***	1.86 (1.049, 3.32)*
	No	62	145	1	1
Current use of tobacco	Yes	115	86	6.72 (4.14, 10.9)****	1.12 (0.613, 2.07)
	No	52	176	1	1
Positive symptom	> 12	145	57	3.06 (6.54, 7.23)***	3.018 (1.55, 5.84)**
	< 12	56	171	1	1

* p < 0.05, ** p < 0.01, *** p < 0.001 1 Reference Hosmer and Leme show test = 0.734, multi co linearity test (VIF) = 3.5

## Discussion

Risky sexual behavior is the most common behavioral disorder and public health important in the global level. The study found that a number of participants met criteria for risky sexual behavior. The finding of this study revealed that prevalence of risky sexual behavior among schizophrenia patients was 39.4%. Almost thirty-four percent of the male and five percent of female schizophrenia have risky sexual behavior. This study was higher than studies conducted in Turkey which was 26% [9], south India 21% [16] and USA 23% [10]. The possible reason for this variation might be difference in method they used for data collection. Conversely our result is lower than 89% noted in New York [10] and a study conducted in India 51% [17]. The possible reason for the difference might be difference in study design, sample size, data collection tool and cultural difference in study population.

In this study, being male sex the odds of having risky sexual behavior is four times higher as compared to females. This finding is similar with other studies [18–21]. The possible reasons could be having sexual intercourse with multiple non-marital, non-cohabiting sexual partners and not using condoms common in males, substance use were more used among males than females, the third explanation might be due to differences on social dynamics given to sexual behaviors of both gender and nature of illness (schizophrenia) among males.

The odd of having risky sexual behavior was five times higher among ages of 18–24 clients with the rest age group. Possible reasons could be use of substance, psychiatric disorders and risky sexual behaviors both peak in young adulthood and severity of illness have poor prognosis among youth, have a lot of trauma in this age group [22], and low self-esteem and high internal stigma in younger adults with mental illness may cause a failure to provide healthier romantic relationship and associated with failure to advocate for safer sex and their knowledge level about risk sexual behavior and its consequence is unsatisfactory [23–25].

The odd of having risky sexual behavior was two times higher among clients with current alcohol use than not use. This finding is congruent with other studies [8, 26, 27]. Possible explanations were psychoactive substance use and abuse have consistently been found to be associated with sexual risk behavior, effect of alcohol could decrease motivation or ability to have protected sexual intercourse and power to influence an individual’s decision making due to these reasons they could involve in risky sexual behaviors, weighing no damage following the behavior, there is a causal relationship between alcohol and the theoretical determinants of sexual risk behavior, alcohol and risky sexual behavior may be associated at the global level because when people plan to engage in risk behavior they may drink or use drugs to decrease cognitive disagreement about their behavior.

Regarding place of residence; the odds of having risky sexual behavior was seven times higher among clients live in urban than living in rural area. It might be due to prostitution, homelessness more common in urban than rural area like other studies [28]. Participants with severe positive symptoms were three times more likely to engage in risky sexual behaviors compared to those without severe positive symptom. This finding is similar with a previous study [7]. A possible explanation is patients who have positive symptoms are self-destructive, having rational thinking loss, maybe less likely to inhibit their sexual impulses, so they do not care about life itself, there is no reason to care whether they acquire an infection or not.

### Conclusion

The prevalence of risky sexual behavior was found to be high. Emphasis should be given for clients who are male, those found at a young age, who are current alcohol user, who live in urban and those present with severe positive symptom. Interventions should contain widespread sexual and reproductive health awareness on issues such as sexual education, safe sex and sexually transmitted infections for clients who are schizophrenic.

## Limitations of the study

Cross-sectional study does not always confirm a cause-and-effect relationship.

## Data Availability

All relevant data are within the paper.

## Abbreviations

OR: odd ratio
COR: crude odd ratio
AOR: adjusted odd ratio
PANNS: positive and negative syndrome scale
